# In-Flight Electromagnetic Compatibility of Airborne Vertical VLF Antennas

**DOI:** 10.3390/s22145302

**Published:** 2022-07-15

**Authors:** Tomasz Aleksander Miś, Józef Modelski

**Affiliations:** Warsaw University of Technology, Institute of Radioelectronics and Multimedia Technology, ul. Nowowiejska 15/19, 00-665 Warszawa, Poland; jozef.modelski@pw.edu.pl

**Keywords:** VLF, linear antenna, stratosphere, balloon, EM compatibility, corona, discharge, cloud, remote sensing

## Abstract

Long-wire very low frequency antennas, when lifted up on high altitudes by an aerostat, move through different atmospheric layers and interact with them electrically in a more intense way in comparison with aircraft flights. Such interactions manifest themselves in the form of electrical changes in the clouds and corona discharges excited on the antenna wire, which may increase the risk of mechanical damages and transmitter overload. In order to investigate the interactions between the different types of clouds and a long balloon-borne antenna wire, two theoretical models were developed and compared with results from an experimental balloon flight directly through a storm front. Based on the theoretical and experimental results, the most accurate model proposed was chosen, as well as a set of basic requirements for the balloon-borne VLF antenna system, reducing the risk of failure during operation in highly electrically active atmospheric environments.

## 1. Introduction

Very low radio frequencies (VLF, 3–30 kHz) have been used since the 1920s for long-range stable telegraphic communication, transmission of time- and frequency standards, and communicating with submerged submarines and mines. Antenna systems required to efficiently emit signals in these frequency range are, for mechanical and maintenance reasons, often reduced to multiple tuned mast-antenna systems with capacitive overhead wire nets [1]. The overall linear dimensions of the radiating structures of these systems are designed to be as close as possible to the order of magnitude of the emitted wavelengths—tens of kilometers. The elaborated mechanical structures pose significant costs of construction and periodic maintenance [2], which are difficult to handle if the designed system is to be employed occasionally, for scientific purposes, or as a support for existing stations. The actual sizes of the VLF antenna systems also make them easily traceable on satellite and radar data, which—in addition to their strategic importance—poses a threat of becoming an easy target for enemy activities.

Solutions to reduce the costs and resources needed to establish a fully operating VLF antenna system are either supporting the ground-anchored radiating structure (wire) by an aerostat [3] or creating a fully airborne antenna system, lifted up to higher altitudes by an airplane or an aerostat [4,5]—not only dismissing the requirement of large mechanical supporting structures on ground, but also—if a stratospheric (reaching the altitude above ~12 km) balloon is employed—moving the VLF transmitting system into an environment less accessible to conventional aircraft and weapons (low temperatures and low partial pressure of oxygen, impeding the engines’ combustion processes), making it essentially unobtrusive and naturally protected. It shall also allow the use of such VLF transmitters for remote sensing of the atmosphere/clouds and ground layers, similarly to magnetic ferrite-core loop antennas lifted up by helicopters [6]. In comparison with horizontal antennas trailed by conventional aircraft, balloon-borne systems also consume less resources (fuel and maintenance costs), provide more stationary operation of the system with less risk from extensive mechanical loads due to flight dynamics, offer greater radiation efficiencies (the elongation of the radiator is not problematic), achieve wider signal coverages, and require less power for operation.

Balloon-borne antenna systems move through the atmosphere with total velocities much lower than these of an airplane (as the latter, being an aerodyne, requires a high velocity to create the lifting forces), consuming less energy, and allowing the use of vertical linear antennas, providing an effective mean to emit a vertically polarized signal that possesses superior characteristics when exciting the terrestrial (ground–ionosphere) waveguide [7,8].

Such systems, however, interact with the surrounding atmosphere differently than the horizontal aircraft-trailed wires, as the balloon-lifted vertical wires are not only subjected to different speeds of electric charge accumulation and loss, but also may act as a shunt between two differently charged regions of a cloud, the flight through which cannot be directly avoided (as the ground-bound balloon pilot—used if a heavy balloon mission is flown—cannot change the mission’s trajectory, except for the remote forced initiation of the descent phase). All of these phenomena lead to the potential occurrence of intense corona and electrical discharges, which could damage the antenna itself, its coupling elements, and the transmitter [9]; additional requirements and constraints must be issued for the system in order to protect it from direct damage from electrical overloads and mechanical damages resulting from interactions with the atmospheric electricity. It is of great importance to investigate the electrical compatibility of such balloon missions—this type of elaborated analysis often lacks in antenna design, and problems linked to electrostatic interactions of the craft materials with the atmosphere and clouds have already caused serious accidents in lighter-than-air aviation (e.g., the catastrophes of the LZ129 Hindenburg or the Star of Poland Stratostat [10]).

Modern research [11,12,13,14,15] concentrates mainly on the electrification of the clouds themselves (including assumptions on the future/possible numerical models), electrification and discharges on plastics, and the occurrence and properties of lightning strikes on aircraft (natural, artificial, and model setups). The acquisition and loss of electric charge in aircraft was addressed in Reference [15]; however, no cloud-related mechanisms were presented, as the major focus was on the inception of lightning. An optional solution to assess the electric charge loss was based on the measurement of the lightnings’ lengths, which required experimental optical data. As the experimental case considered in this paper is an atypical one (slow vertical metallic airborne setup with low velocities), new formulations must be created by using adapted basic formulas and then concatenated to give the first complex descriptions of these electrostatic interactions. In this paper, such interactions—e.g., the increase of electric field strength around the large air-floating antenna—are modeled by using two different models of interaction with clouds and their proximities; these models are then compared with direct corona and discharge data obtained from a rare case of a stratospheric flight directly through a warm storm front. Finally, a set of additional requirements and constraints is presented in order to protect the transmitting balloon-borne system from direct damage.

## 2. Atmospheric Electricity Basics

The Earth’s atmosphere remains rich in ions of different charges produced by various physical phenomena—UV radiation, natural radioactivity, ionization in electric fields, etc. [11]; the planet itself has a total negative electric charge [16]. The formation, maturation, and dispersion of clouds is largely dependent on the electrostatic interactions between the atmospheric ions, water droplets, and the surrounding air (a cloud cannot be considered as a static-like ‘resistance’ in the ground–ionosphere circuit—it acts as an active source of electric charge [17])—any object traveling in this space is also subjected to these mechanisms, albeit in different speed (charge accumulation and loss) and magnitude.

### 2.1. Atmospheric Loss of Electric Charge

The clouds, due to the processes of coagulation of water droplets which capture free-floating ions, experience a very low electrical resistance, lower than the air in good weather. The conductivity of the air increases with altitude, reaching the highest values in the ionosphere. The precipitation (rain) leaving the cloud possesses a positive charge shortly after exiting the cloud; later on, however, during the fall, the charge drops (gets mixed, positive and negative) due to electrostatic interactions between the individual droplets of different sizes and the surrounding air [17].

Due to the significant difference in electrical resistances between the cloud and the air in good weather, the charged particle/object loses its electric charge much faster in the latter conditions. This process is exponential and accelerates with the increase of altitude; the general function of this decrease from the particle’s initial electric charge *Q*_0_ (C) can be formulated as follows:

(1) $$Q_{loss}=Q_{0}e^{-\frac{1}{\tau_{loss}}t}$$

where the time constant, *τ_loss_* (s), corresponds to the time after which the electric charge of a particle decreases three times in the cloudless air [13]. This constant is a function of altitude *H* (m) and can be approximated for different altitude ranges by using the given formulas:

(2) $$\tau_{loss}=728.19e^{-5\cdot 10^{-4}\cdot H}\;\wedge \;0\;m<H\leq 2000\;m$$

(3) $$\tau_{loss}=535.5e^{-3\cdot 10^{-4}\cdot H}\;\wedge \;2000\;m<H\leq 4000\;m$$

(4) $$\tau_{loss}=360.8e^{-3\cdot 10^{-4}\cdot H}\;\wedge \;4000\;m<H\leq 6000\;m$$

(5) $$\tau_{loss}=272.1e^{-2\cdot 10^{-4}\cdot H}\;\wedge \;6000\;m<H\leq 8000\;m$$

In the presence of high electric fields and/or large electric charges, the loss of electric charge of an object may manifest itself in the form of corona—short electrical discharges, not always accompanied by glowing ionization of the air, appearing rapidly with clear intervals. The electric field strength above which the corona appears—the corona initiation field strength—decreases exponentially with the altitude, following Paschen’s law describing the breakdown voltage/electric field strength as a function of air pressure [18,19]; according to the research on electrostatic precipitators (industrial devices used to electrically separate different dust fractions by electrifying them), the corona is more easily initiated on wires of larger radii (larger surface) [20]—this is consistent with the experiments on aircraft-trailed wires, which exhibited corona of increasing intensity with the increase of wire length [21]. Atmospheric experiments on the appearance of corona showed that, for higher altitudes—lower air pressure—the corona initiation field strength varies from 7.2 kV/cm for the altitude of 18,300 m to 4.6 kV/cm for 30,500 m; laboratory experiments with charged dielectrics (Mylar sheets) showed that the Paschen’s law indicated the surface charge density of the charged object (surface charged density needed for the breakdown to occur) as one degree of magnitude higher than in the actual experiments [12].

**Figure 1:**
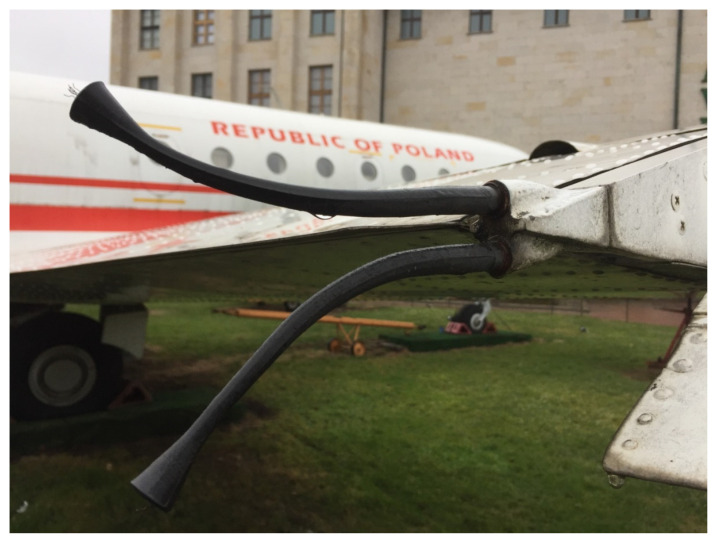
Electrostatic dischargers permanently riveted onto the rear part of the wing of the Yakovlev Yak-40 no. 044 (Polish Air Force); the rubber insulation, expanding at the end, moves away from the corona-susceptible wire bundles from the aircraft’s structure; the wire bundles are easily noticeable at the ends of the cables. The airplane has such dischargers affixed on both wings and on the upper part of the vertical stabilizer.

For the airplanes, the loss of electric charge acquired via friction with the water and ice particles, as well as by the electrostatic induction, is facilitated by the affixation of special dischargers, consisting of an externally insulated short cable with a bundle of thin wires at the end [21]. The dischargers are affixed on the extremities of the aircraft (wing tips, winglets, and tips of the vertical and horizontal stabilizers), where the electric charge density is maximized, accelerating the charge loss (see Figure 1). As the aircraft moves with the average velocities of hundreds of km/h through electrically charged regions in the atmosphere, its presence in these regions is too short to actively influence the clouds’ structures and evolution. Slowly moving balloon missions are subjected to slower acquisition of electric charge by friction with surrounding particles, but, due to the much longer presence in the clouds (due to slower movement), they may be considered to be a disturbance of the cloud’s homogeneity, influencing its evolution and the surrounding electric field.

### 2.2. Electric Fields in Clouds

**Figure 2:**
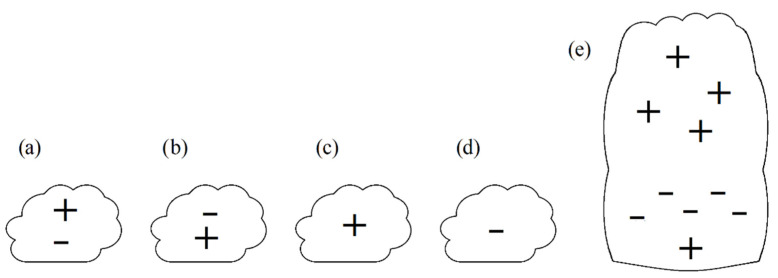
The schematics of basic electrical structures encountered in horizontal layered clouds: (**a**) positive polarization, (**b**) negative polarization, (**c**) total positive charge, (**d**) total negative charge, and (**e**) for comparison—electrical structure of a towering vertical storm cloud.

A cloudless atmosphere experiences a natural electric field of approximately 1 V/cm on the ground’s surface, dropping exponentially to 0.2 V/cm above 2 km of altitude and below 0.03 V/cm above 9 km of altitude [22]. This weak electric field is considered to be a primordial cause for the creation of a double-polarized layered zone of aerosols and ions, producing a double-charged cloud. This, however, is not the only encountered type of cloud; Figure 2 presents the four basic types of electrical structures of a cloud, compared with the electrical structure of a storm, towering vertical cloud [17].

**Figure 3:**
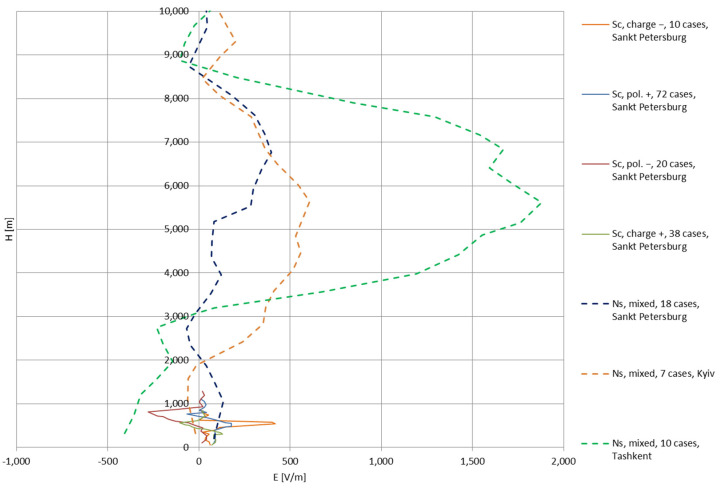
The electric field strengths measured for different types of cloud polarizations (data based on [17]).

Imianitov et al. [17] presented exemplary data on the measured values of the electric field strength in two types of clouds—Stratocumulus (Sc, layered, rainless) and Nimbostratus (Ns, matured storm cloud, a component of heavy-rain storm fronts) [16]. The Sc measurements were taken in Sankt Petersburg in 1958–1959 for all four types of cloud polarizations depicted in Figure 2 (in total 140 cases); the Ns measurements were taken in Sankt Petersburg in 1960–1962, Kyiv in 1960–1963, and Tashkent in 1960–1961 (in total 35 cases). Figure 3 presents the measured electric field strength values for varying altitude; the Sc clouds occupy altitudes below 1 km and show significantly lower values of electric field strengths. This, however, does not necessarily indicate lower intensities of electrical charging and charge maintenance on floating objects, as the conductivity of the air in this region is much lower than above the Ns clouds.

## 3. Antenna In-Cloud Charging Mechanisms

**Figure 4:**
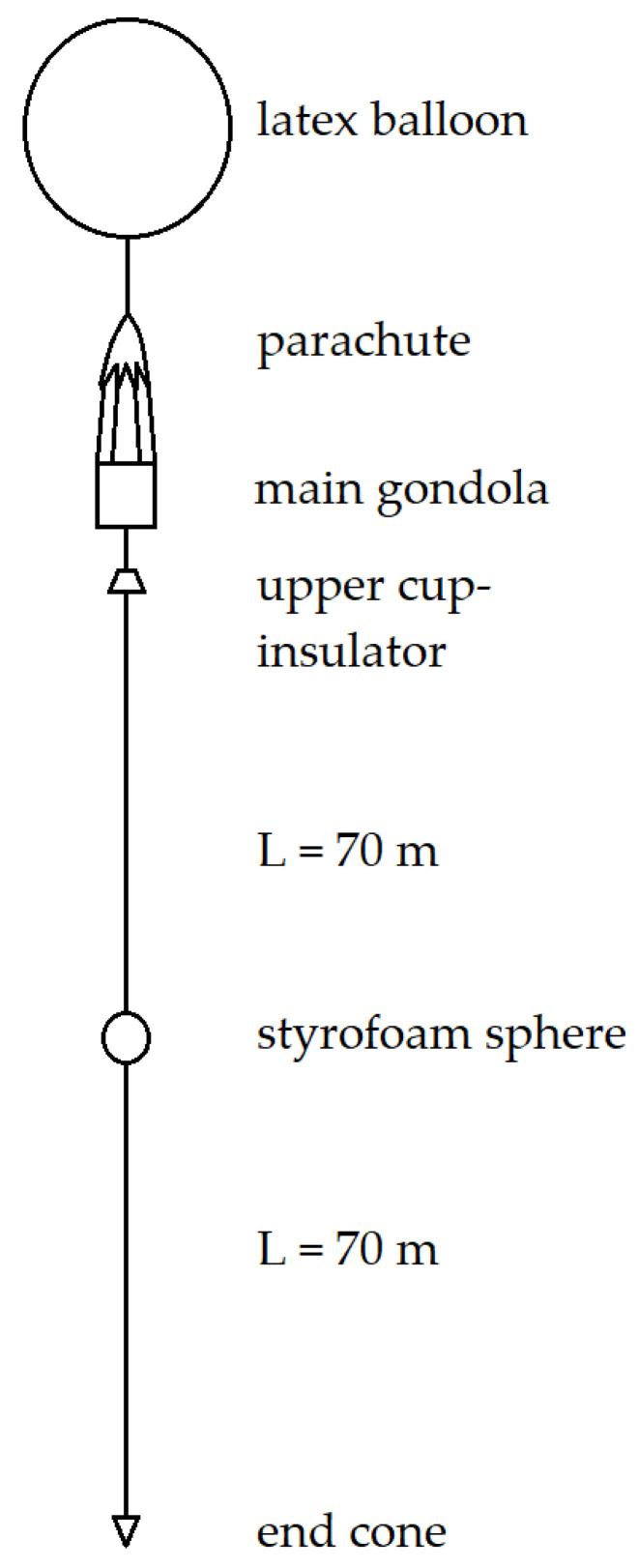
The general schematic of the balloon mission used in the analysis. The antenna wire lengths are not to scale. The schematic does not include small red flags made of non-conductive fabric, placed evenly on the antenna to provide enhanced visibility.

The balloon-borne VLF antenna in this analysis—both theoretical and experimental —has the form of a tape-like flat aluminum wire having total length *L* of 140 m, width *b* of 15 mm, and thickness *d* of 0.1 mm, with one side covered with plastic-coated paper (Figure 4). The electrostatic interactions with the atmosphere are related to the surface of the conducting object, so, in this case, the following formulas (originally derived for small objects [17]) refer to the area *L·d* (m^2^), with the assumption that the physical phenomena responsible for the accumulation and loss of electric charge remain the same.

### 3.1. Static Electric Field Model

During the ascent phase, the balloon mission, with a long vertical antenna, travels through three basic types of regions—cloudless, electric field in proximity to the cloud, and electric field inside the cloud.

The cloudless zone exhibits electric charge exponential loss with the time constant, τ_loss_, dependent on the altitude, as defined by Formula (1).

The zone with electric field in proximity to the cloud—with particle density lower than inside the cloud—exhibits both electric charge accumulation and loss, with the maximum electric charge, *Q_max_.* (C) formulated as follows [13]:

(6) $$Q_{max.}=0.515\varepsilon_{0}ELd$$

where ε_0_ ≈ 8.854 F/m is the electrical permittivity of vacuum, and *E* (V/m) is the external electric field intensity. This maximum value is reached exponentially in time, with the time constant derived from the experimentally defined charge accumulation rate, *v_C_* (in this analysis, equal to 0.3 × 10^−13^ C/m^3^/s [17], and 10^4^ times more for the storm cloud conditions):

(7) $$\tau =\frac{Q_{max.}}{Ldbv_{C}}$$

Therefore, the electric charge accumulation and loss in this region can be defined as follows:

(8) $$Q_{I}\left(t\right)=0.515\varepsilon_{0}ELd\left(1-e^{-\frac{bv_{C}}{0.515\varepsilon_{0}E}t}+e^{-\frac{1}{\tau_{loss}}t}\right)$$

The definition of electric field strength at a given distance r (m) from the wire remains under an assumption that the distance, *r*, is defined between the wire and an imaginative second electrode in the cloud or surrounding air (as there are no such electrodes in real experiments [12]). The electric potential distribution around the analyzed wire can be defined as follows [23]:

(9) $$V=\frac{Q_{max.}}{4\pi \varepsilon_{0}Ld}\log\left(\frac{a+c}{a-c}\right)\;$$

(10) $$a=\sqrt{r^{2}+c^{2}}\;$$

where *c* is the half of the wire’s length (here equal to 70 m), and distance r is assumed at 1 m. In real conditions, the distance, *r*, is a variable dependent on the actual shape of the cloud and the position of the equipment in relation to the cloud’s electric charge concentrations (if assumed that they could be considered as the ‘2nd electrodes’). The exemplary experimental electric field strength data used in this comparison do not indicate any more information related to the geometry of the measuring equipment/environment (e.g., the distance between the probes), presenting the electric field strength values only. If the aforementioned assumption is maintained and a simulated (or experimental) cloud shape is delivered, a distance, r, could be introduced as a defined non-unitary value; however, this would limit the usefulness of the calculations, as the considered clouds present a lack of repeatability in this geometric scale (except their general types and substructures). Hence, the unitary value of the distance, r, was assumed.

By using this formula, the electric field intensity, *E_I_* (V/m), around the electrified wire in this region can be derived:

(11) $$E_{I}=\frac{0.515E}{4\pi r}\left(log\frac{a+c}{a-c}\right)\left(1-e^{-\frac{bv_{C}}{0.515\varepsilon_{0}E}t}+e^{-\frac{1}{\tau_{loss}}t}\right)$$

The third zone—the inside of the cloud—can be characterized by an increased rate of electric charge accumulation and negligible charge loss; thus, Formula (8) is reformulated into the following [17]:

(12) $$Q_{II}\left(t\right)=3\varepsilon_{0}ELd\left(1-e^{-\frac{bv_{C}}{3\varepsilon_{0}E}t}\right)\;$$

resulting in the respective formula for the electric field strength, *E_II_* (V/m):

(13) $$E_{II}=\frac{3E}{4\pi r}\left(log\frac{a+c}{a-c}\right)\left(1-e^{-\frac{bv_{C}}{0.515\varepsilon_{0}E}t}\right)\;$$

The external electric field strength, E, can be taken directly from the experimental data shown in Figure 3; as these functions are altitude-dependent, to form such dependence in Formulas (12) and (13), the time, *t* (s), can be substituted with a ratio *H/v_Z_*, where *H* (m) is the altitude, and *v_Z_* (m/s) is the ascent rate of the balloon mission (chosen arbitrarily or based on the actual flight data).

Additional elements of the electrification process of the balloon-borne wire are the electric charge acquisition during the takeoff procedure and the creation of potential difference during the freezing of remains of liquid water remaining on the system.

As the wire antenna of the balloon mission is lifted up from the ground from a lying-down, already-deployed position, it remains in a rubbing contact with the partially conductive substrate (the airfield—humid grassy surface). As the antenna rises, a difference of electric potential appears as an effect of the separation of charges between the wire and the ground [24]; assuming that the dielectric compound of the aluminum antenna (the supportive substrate of the wire) is partially composed of polyethylene, the maximum electric field strength resulting from this mechanism can be approximated as +300 V/m (lower than the mean value for different types of polyethylene due to expected irregularities in the process from the rugged airfield surface) [25].

In the mid-1940s, the works of Workman and Reynolds, as well as Gunn and Dinger, showed that, during the freezing process of water, an increase of the electric potential can be observed [26]; this process is able to produce electrical currents as high as 1 microampere and is experienced only during the freezing (does not manifest itself after the freezing stops) [27]. In the current analysis, the freezing of possible water that remains accumulated on the antenna system may occur in the Ns clouds at the altitude of the 0 °C isotherm, around the altitude of 4.2 km from the data set in Figure 3. The maximum electric potential difference reached in this manner is +230 V (ice positive) [26,27].

The ‘static electric field model’ approach mentioned in the title of this section means that it is assumed here that the balloon mission does not interfere with the electrical structure of the clouds, thus allowing the use of encountered external electric field strength, E, in Formulas (11) and (13) (as presented in Figure 3). This does not necessarily correspond to the reality, as the balloon mission moves through the atmosphere with velocities low enough for it to be considered as a disturbance in the cloud’s structure, which is theoretically able to modify its composition and the existing electric field (as mentioned in Section 2.1). Therefore, for comparative purposes, a ‘dynamic electric field model’ is proposed below.

### 3.2. Dynamic Electric Field Model

The basis for the definition of the possible influence of the slow-moving balloon mission with a long vertical antenna on the electric field in the cloud and its proximities is the assumption that, during the passage of the balloon mission, the cloud remains in a quasi-constant state, which can be described by the equation of cloud equilibrium [17]:

(14) $$E_{below\;cloud}\lambda_{below\;cloud}=E_{cloud}\lambda_{cloud}=E_{above\;cloud}\lambda_{above\;cloud}\;$$

which describes the current densities (A/m^2^) in different electrified regions, with *E* (V/m) defined as previously, and *λ* (S/m) as the conductivity of the given region.

The three electrified regions—below the cloud, inside the cloud, and above the cloud —physically do not exist as a discontinuous formation; that is, the passage from one zone to another is smooth and continuous. Therefore, if discretized for different adjacent altitudes, H, the equation takes the following form:

(15) $$E_{H-1}\lambda_{H-1}=E_{H}\lambda_{H}=E_{H+1}\lambda_{H+1}\;$$

If the adjacent step’s index is changed from *H* to a common *i*, *E′* is introduced as the value of the electric field strength modified by the presence of the antenna wire, and the index *A* is introduced as the one describing a parameter related to the antenna wire itself. Thus, Formula (15) can be expanded as follows:

(16) $$\left(E_{i}+E_{A}\right)\left(\lambda_{i}+\alpha \lambda_{A}\right)=E_{i+1}^{’}\left(\lambda_{i+1}+\alpha \lambda_{A}\right)\;$$

where α (–) is the ratio of the antenna wire volume to the volume surrounding it with the boundary of *r* = 1 m (similarly to the assumption in the previous section). The modified electric field strength, *E′*, in the next (*i + 1*) step can be easily calculated from this formula; this value shall be then used in Formulas (11) and (13). The mode of employment of Formula (16) is recursive; that is, the antenna electric fields calculated from (11) and (13) become *E_A_* in the next calculation step (next altitude value), along with the respective *E_i_* value from the cloud (Figure 3).

The values of the conductivities in the clouds can be calculated by using the experimental electric field values and the current densities—for the Ns clouds, under a macroscopic (large area) approach the current density reaches 10^−7^ A/m^2^; for the Sc clouds, assuming that the maximum conductivity may reach 1/25 of the conductivity of clear air (mean clear air value: 1.5 × 10^−14^ S/m [28]), the current densities in the central part of the Sc clouds reach values between 0.7 × 10^−13^ and 2.5 × 10^−13^ A/m^2^ [17].

### 3.3. Theoretical Simulation Results

To simulate the gain and/or loss of electric field strength of a balloon-born vertical wire (dimensions given in Section 3), the calculations were performed for two cases—‘static’ and ‘dynamic’ (or influenced)—using data points (*H*; *E*) plotted in Figure 3. The altitude axes were divided into subsequent calculation stages, or zones, where specific phenomena of electric charge accumulation and loss appear:

- Zone I: charge loss in clear air—Formulas (1) and (2), charge accumulation during takeoff, resulting in initial rise of electric potential (expanded to two cases: +300 V and −300 V),
- Zone II: charge loss and accumulation in the proximity to the cloud—Formula (11),
- Zone III: charge accumulation in the cloud—Formula (13); for the Ns clouds, the additional short local increase of electric potential of +230 V due to freezing of possible water remains at the 0 °C isotherm,
- Zone IV: charge accumulation and loss above the cloud (with non-negligible external electric field strength), approximated by Formula (11),
- Zone V: charge loss in clear air—Formulas (1) and (2)–(5)—depending on the upper borderline of the electric field occurrence.

The ‘dynamic’ cases were expanded by using Formula (16), as described above. In all cases, the *v_Z_* (ascent velocity) was defined as 3 m/s (consistent with experimental flight readings); the parameters of the antenna wire are used as defined in Section 3 (*L* = 140 m). For comparative purposes, a calculation of results for two lengths of wires—10 and 500 m—was also carried out. The calculated electric field strengths were summed with the external electric field strength in order to correspond to the worst-case scenario, where the total electric field strength may appear high enough to induce discharges.

**Figure 5:**
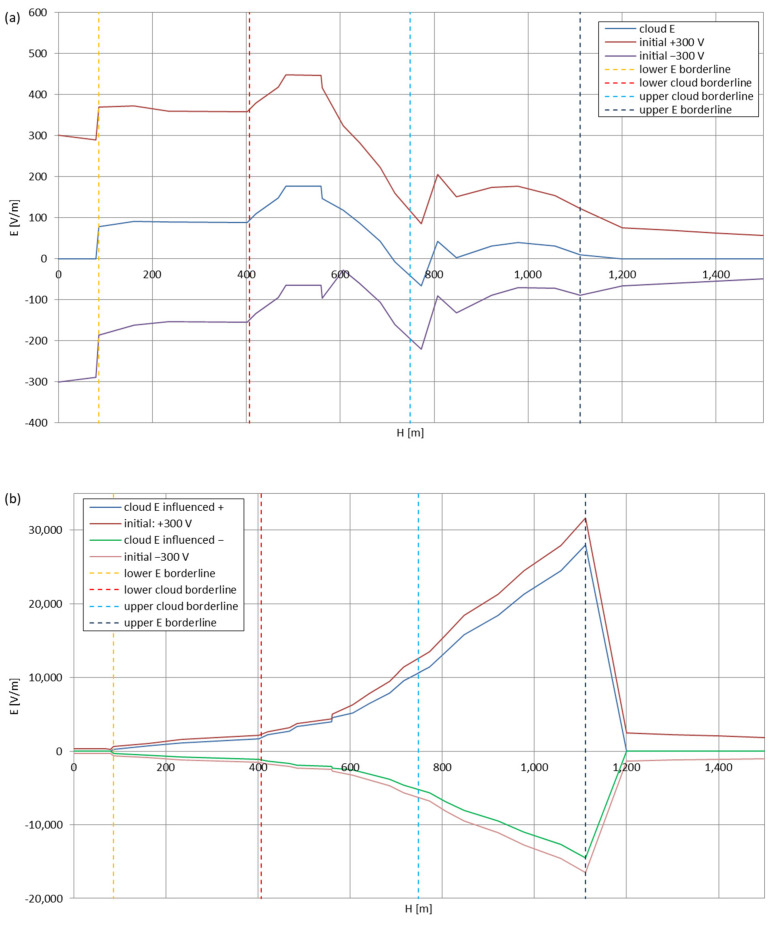
Maximum electric field strengths around the wire antenna passing through Stratocumulus cloud positively polarized: (**a**) ‘static’ case and (**b**) ‘dynamic’ case.

**Figure 6:**
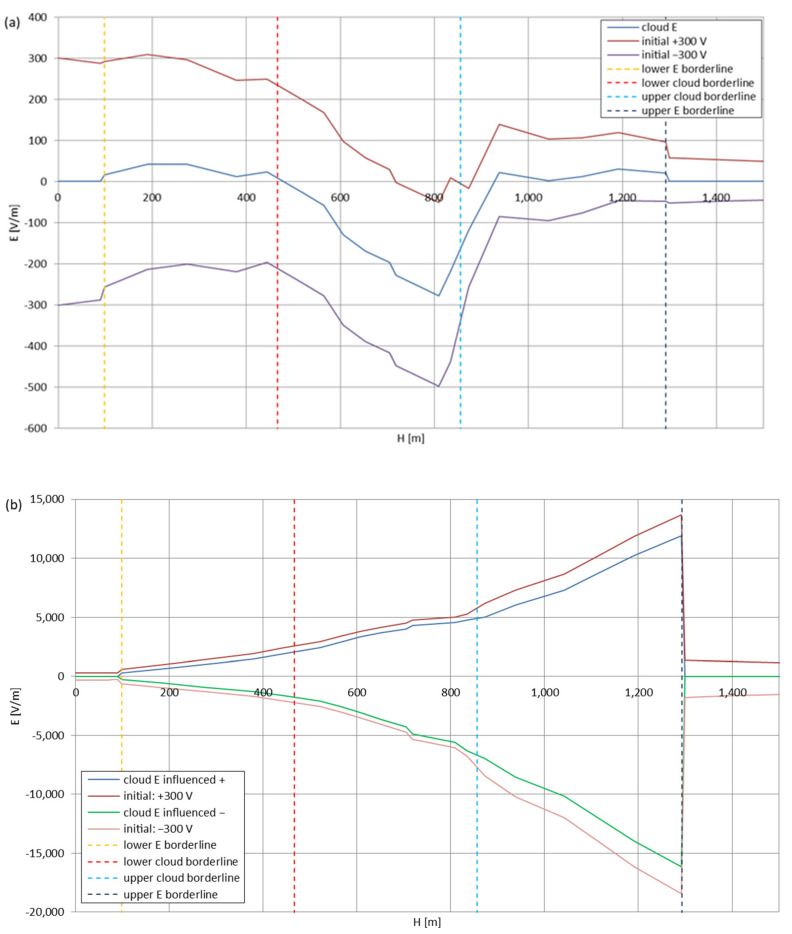
Maximum electric field strengths around the wire antenna passing through Stratocumulus cloud negatively polarized: (**a**) ‘static’ case and (**b**) ‘dynamic’ case.

**Figure 7:**
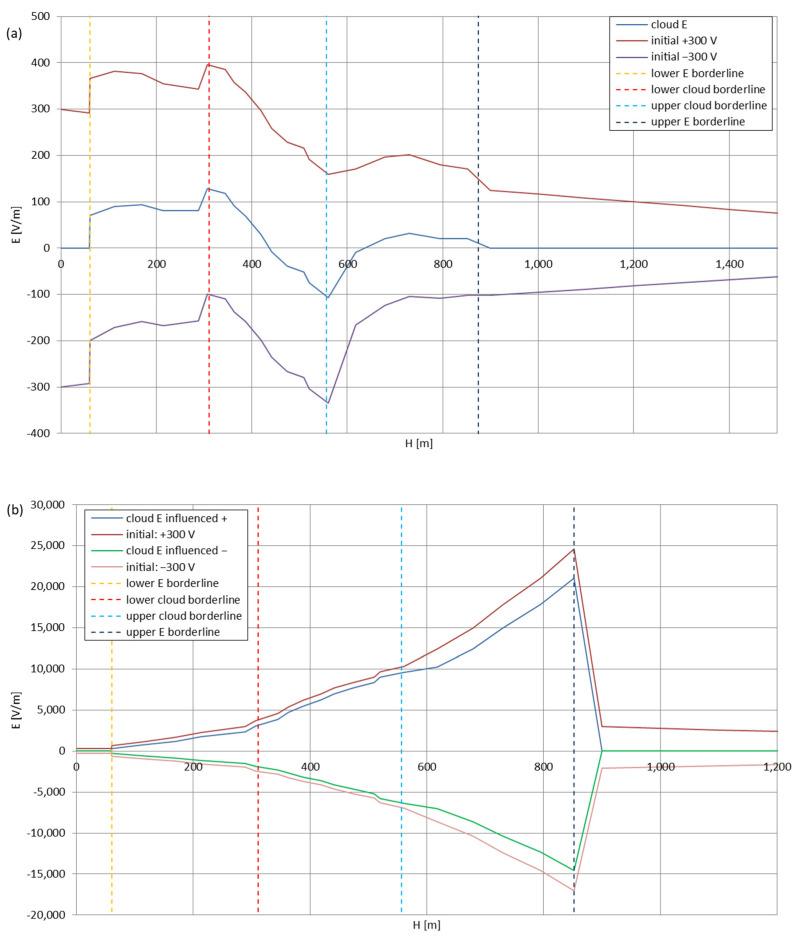
Maximum electric field strengths around the wire antenna passing through Stratocumulus cloud charged positively: (**a**) ‘static’ case and (**b**) ‘dynamic’ case.

**Figure 8:**
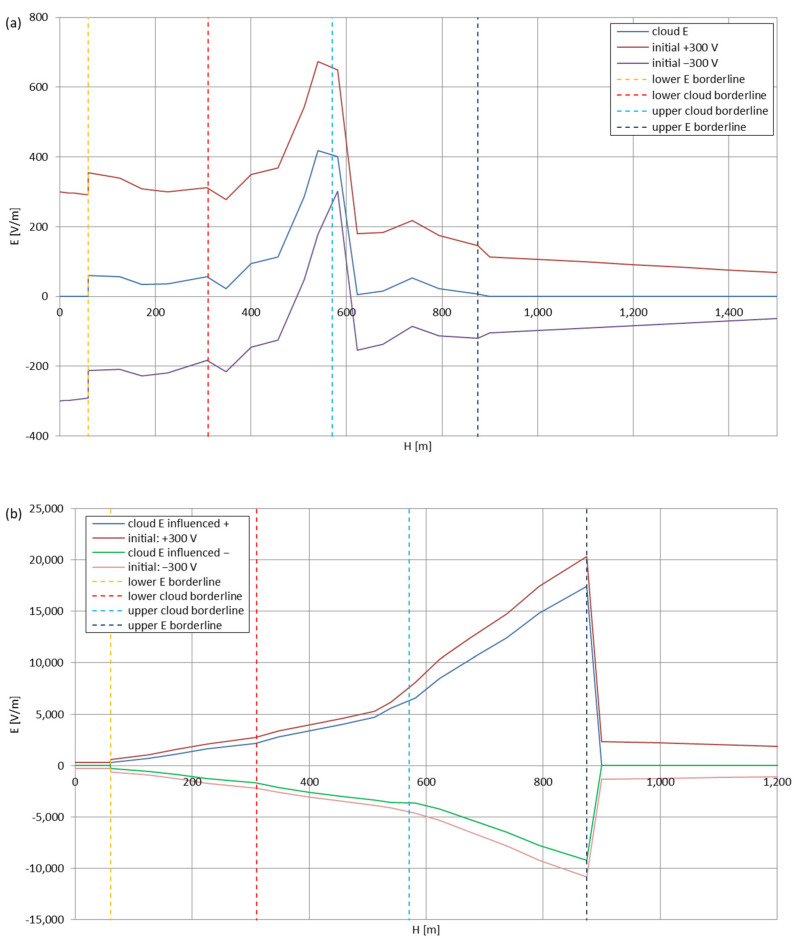
Maximum electric field strengths around the wire antenna passing through Stratocumulus cloud charged negatively: (**a**) ‘static’ case and (**b**) ‘dynamic’ case.

**Figure 9:**
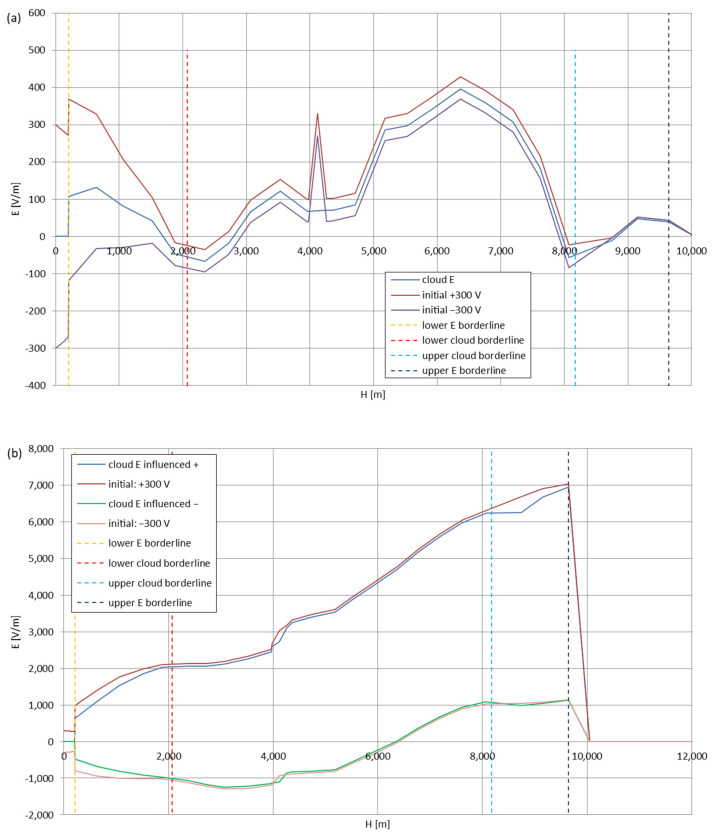
Maximum electric field strengths around the wire antenna passing through Nimbostratus cloud of mixed charge structure, Sankt Petersburg data: (**a**) ‘static’ case and (**b**) ‘dynamic’ case.

**Figure 10:**
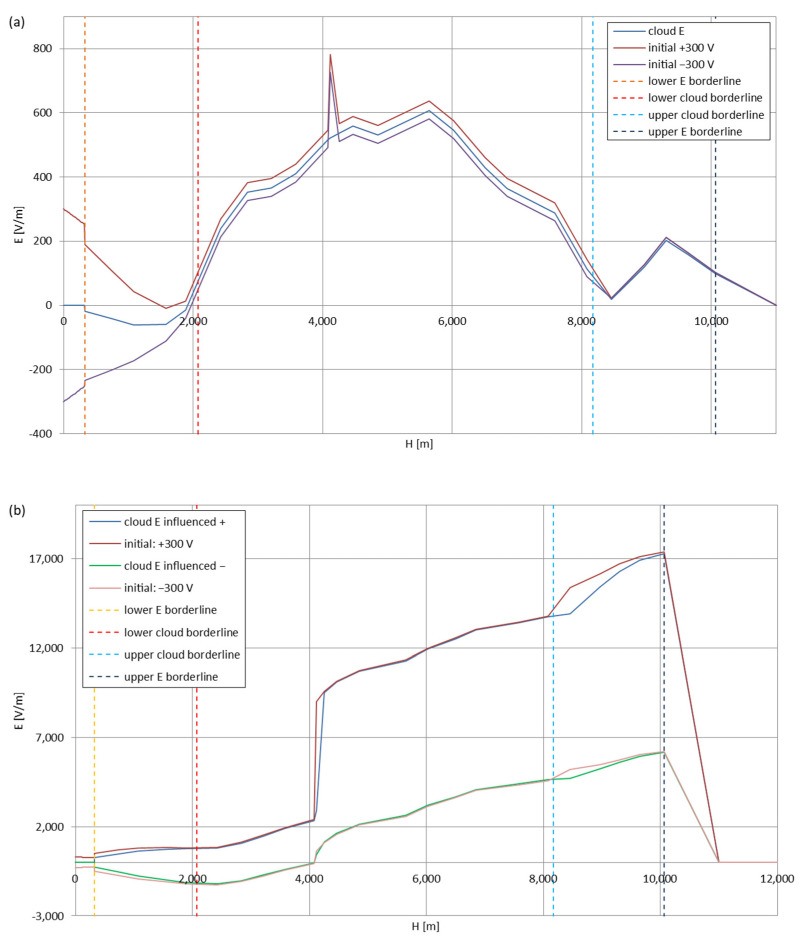
Maximum electric field strengths around the wire antenna passing through Nimbostratus cloud of mixed charge structure, Kyiv data: (**a**) ‘static’ case and (**b**) ‘dynamic’ case.

**Figure 11:**
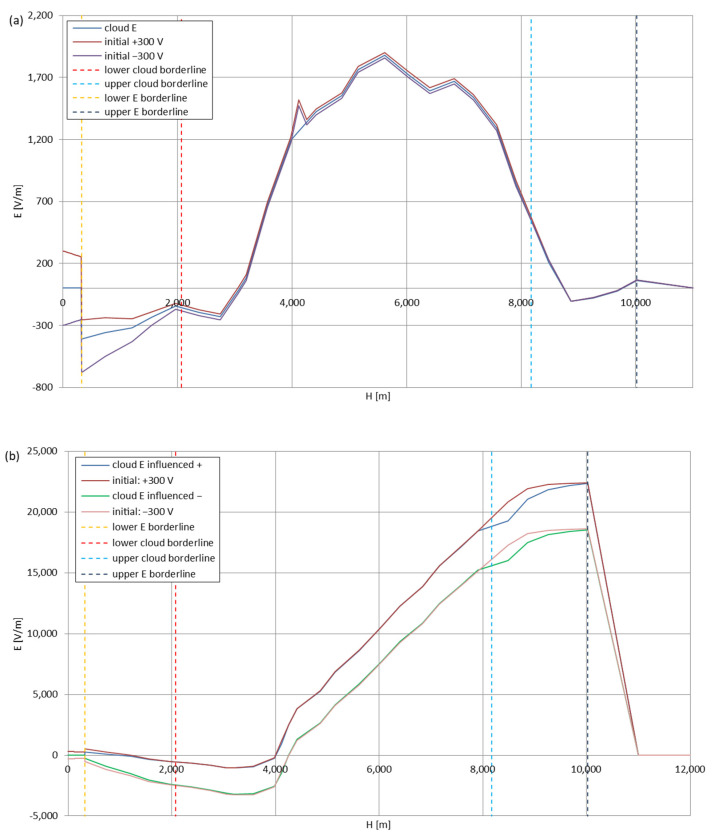
Maximum electric field strengths around the wire antenna passing through Nimbostratus cloud of mixed charge structure, Tashkent data: (**a**) ‘static’ case and (**b**) ‘dynamic’ case.

Figure 5, Figure 6, Figure 7, Figure 8, Figure 9, Figure 10 and Figure 11 present the calculated total electric field strengths for ‘static’ and ‘dynamic’ cases; the functions correspond to the electric field strengths appearing in the moment the antenna passes through the air/cloud.

It can clearly be seen that the maximum values of the electric field strengths for all cases are significantly higher for the ‘dynamic’ cases, where the external electric field in the cloud and in cloud proximities is influenced by a long electrically charged object of different conductivity—the cloud, to maintain its equilibrium according to general Formula (14), for a changing conductivity elevates the electric field strength in the region where the disturbance takes place, as in Formula (16). As the local differences in conductivities reach many degrees of magnitude (air/cloud vs. aluminum), the local electric field strengths are expected to locally increase significantly to compensate for this, according to the assumed aforementioned mechanisms; hence, there are large differences in the E values between the models. For the Sc clouds, the ‘static’ model of electrification presents electric field strengths rarely exceeding 0.5 kV/m, while for the ‘dynamic’ charging model, the values may reach up to 30 kV/m for both Sc and Ns clouds—despite their significant differences in initial electric field strengths, as shown in Figure 3 (this shows that the main causes for the increase of electric charge and electric field strength are the charging mechanisms, not the external electric field itself). The ‘dynamic’ model shows that, for the increasing values of the electric field strength inside the Ns clouds, with the mechanisms of electrification and charge loss adapted to these conditions, the difference between the antenna E values and the cloud’s E values starts to decrease; this effect may be accelerated by the initial course of the cloud’s *E* function. For all cases, the electric field strength drops rapidly after the exit from the zone with external electric field in proximity to the cloud—a result of the increased conductivity of the surrounding air. For the Ns clouds, an increase of the electric field strength can be seen after the exits from the cloud regions (above the upper cloud borderlines)—the effects of changing electric charge accumulation mechanisms. The ‘static’ model clearly shows the electric field strength increase due to freezing of the water residue at the 0 °C isotherm (E value peaks repeating in ‘(a)’ plots shortly above 4000 m of altitude)—this mechanism is perceptible in the ‘dynamic’ model as short increases of the E’s derivatives, which moderately accelerate the electric field strength growth.

**Figure 12:**
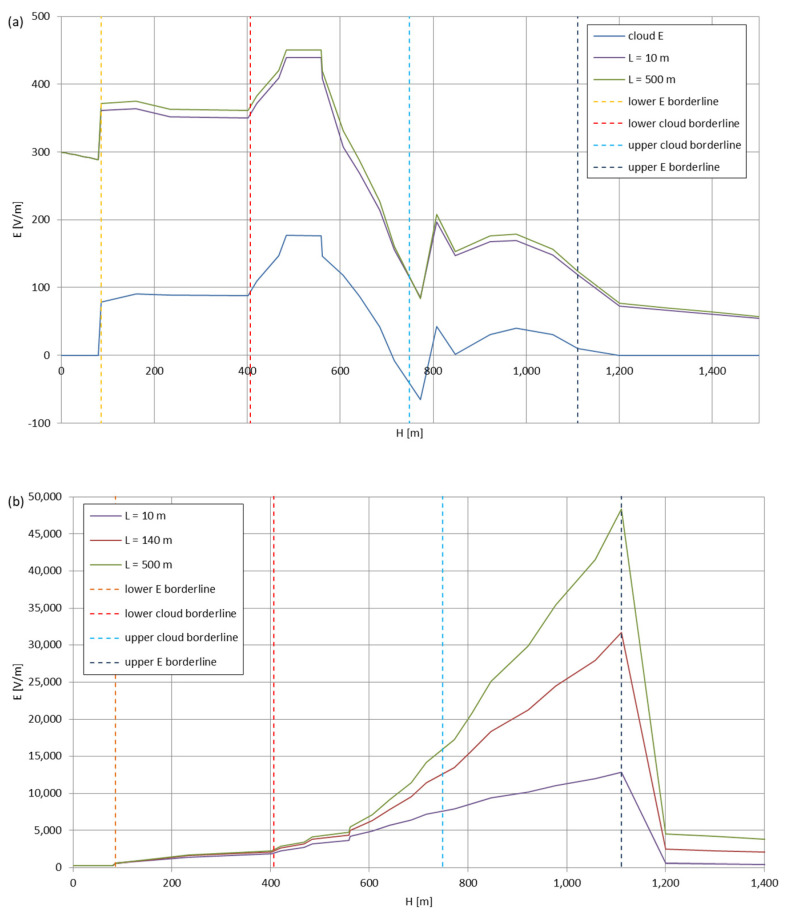
Maximum electric field strengths around the wire antenna passing through Stratocumulus cloud polarized positively: (**a**) ‘static’ case, with the ‘140 m’ data set omitted to enhance curve visibility; and (**b**) ‘dynamic’ case.

Figure 12 presents the comparison for ‘static’ and ‘dynamic’ models for two and three (respectively) wire antenna cases of different lengths for the positively polarized Sc cloud. As expected, the longer wire, in all cases, produces higher electric field strengths (facilitating the occurrence of corona, which is consistent with Reference [21]); the calculations, however, do not include any possible discharging processes resulting from the shunting effect of a wire of that significant length (comparable to the dimensions of the smaller clouds).

**Figure 13:**
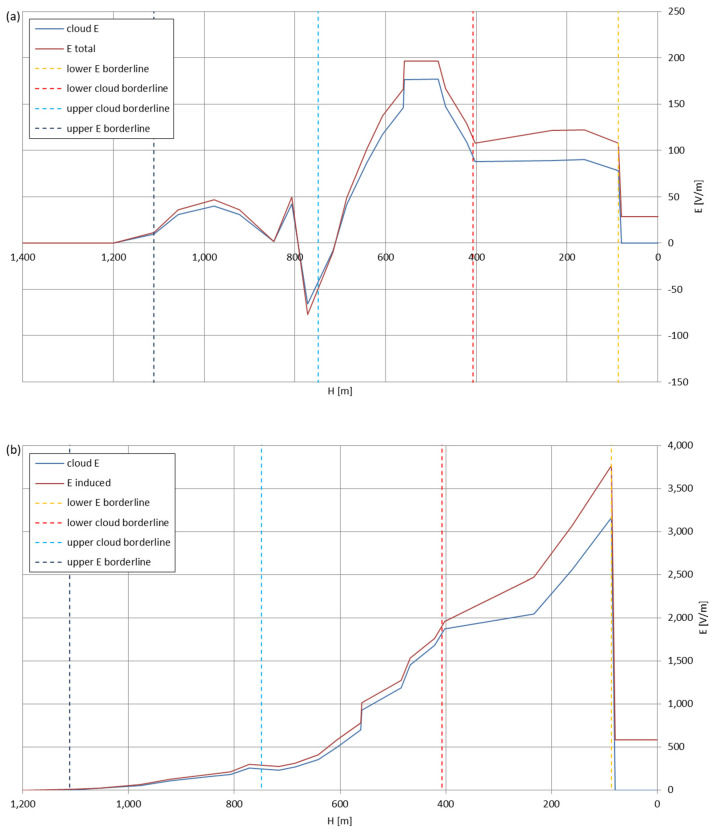
Maximum electric field strengths around the wire antenna re-entering through Stratocumulus cloud positively polarized: (**a**) ‘static’ case and (**b**) ‘dynamic’ case.

**Figure 14:**
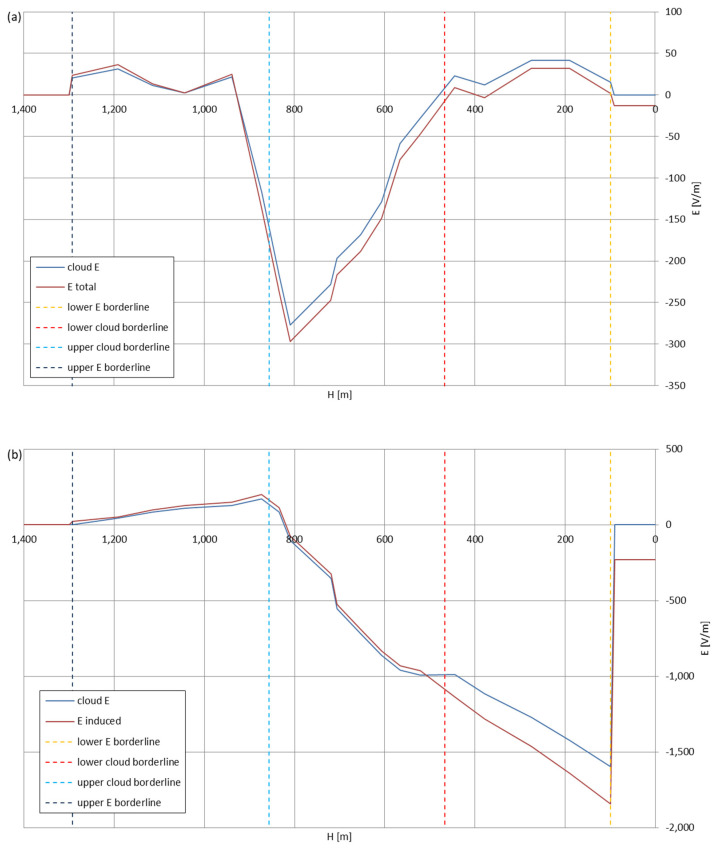
Maximum electric field strengths around the wire antenna re-entering through Stratocumulus cloud negatively polarized: (**a**) ‘static’ case and (**b**) ‘dynamic’ case.

**Figure 15:**
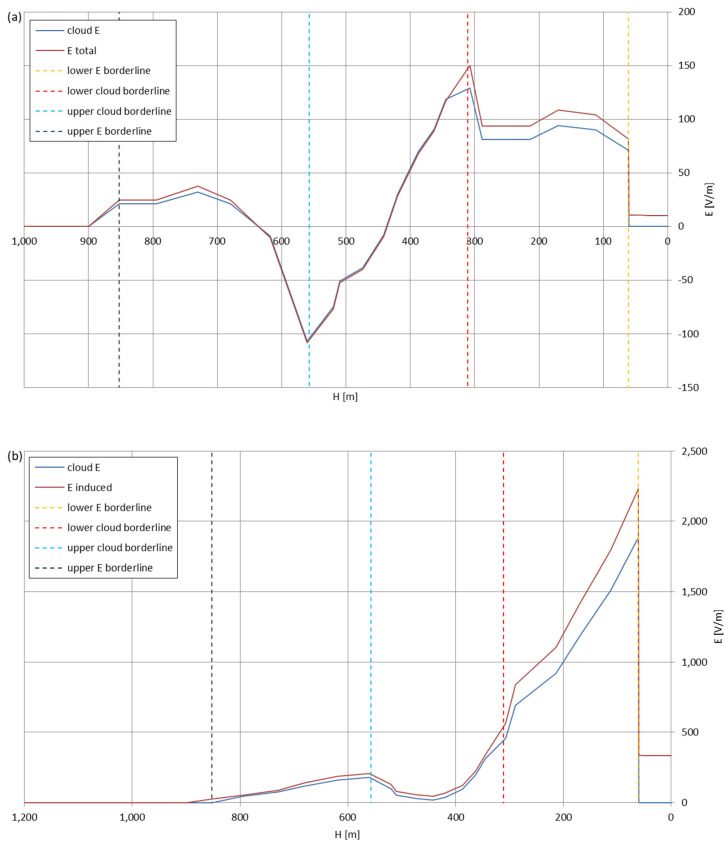
Maximum electric field strengths around the wire antenna re-entering through Stratocumulus cloud charged positively: (**a**) ‘static’ case and (**b**) ‘dynamic’ case.

**Figure 16:**
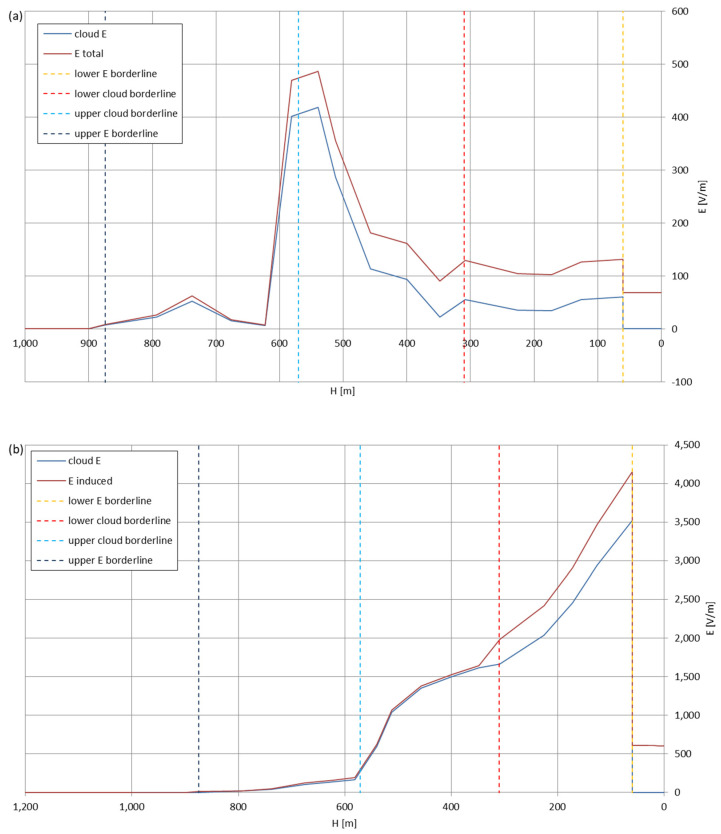
Maximum electric field strengths around the wire antenna re-entering through Stratocumulus cloud charged negatively: (**a**) ‘static’ case and (**b**) ‘dynamic’ case.

**Figure 17:**
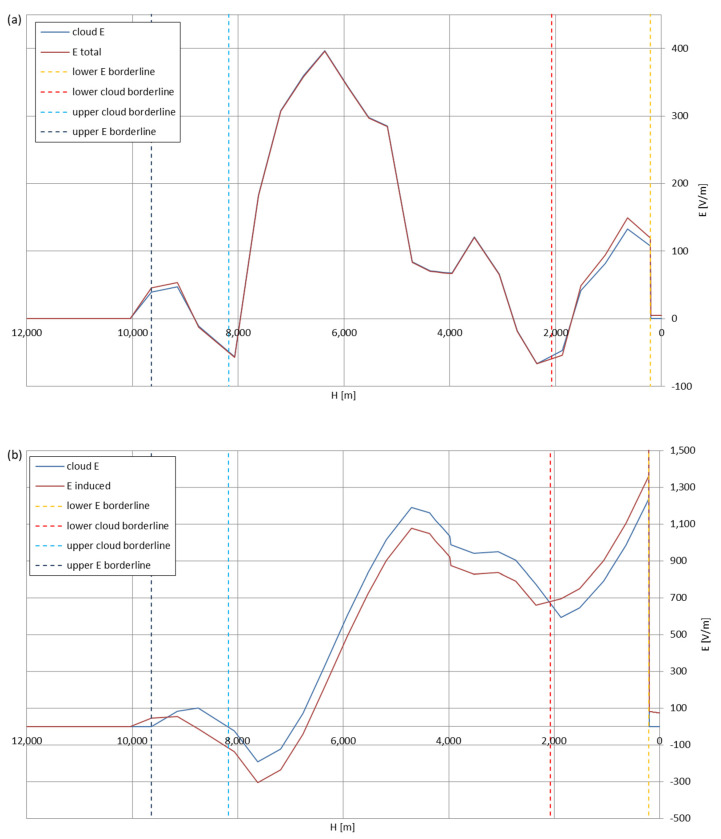
Maximum electric field strengths around the wire antenna re-entering through Nimbostratus cloud of mixed charge structure, Sankt Petersburg data: (**a**) ‘static’ case and (**b**) ‘dynamic’ case.

**Figure 18:**
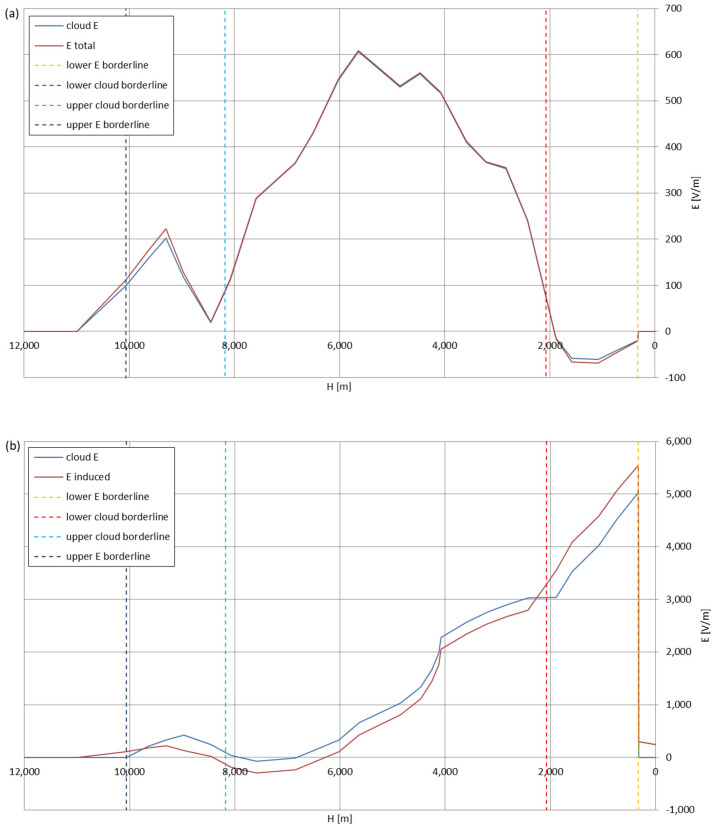
Maximum electric field strengths around the wire antenna re-entering through Nimbostratus cloud of mixed charge structure, Kyiv data: (**a**) ‘static’ case and (**b**) ‘dynamic’ case.

**Figure 19:**
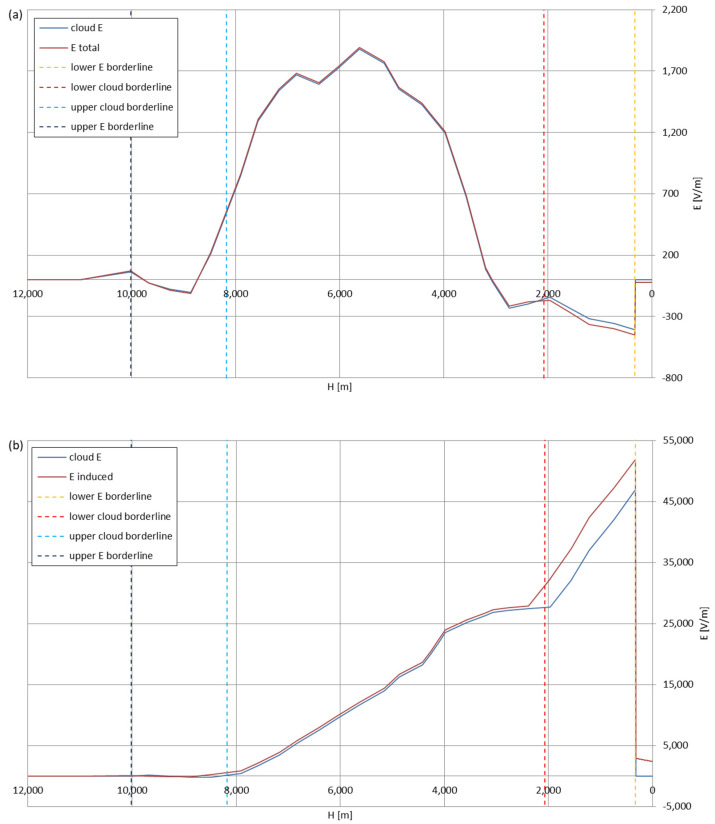
Maximum electric field strengths around the wire antenna re-entering through Nimbostratus cloud of mixed charge structure, Tashkent data: (**a**) ‘static’ case and (**b**) ‘dynamic’ case.

During the parachute-descent phase of the balloon mission—after the balloon explodes at maximum altitude or the payload is deliberately cut off the balloon at target altitude—the antenna, if designed properly, shall also move in a vertical position and enter the cloud with a velocity, v_Z_, approximately five times higher than during the ascent (the velocity, v_Z,_ slowly decreases with altitude, as, with the increasing density of the air, the parachute operates more and more effectively). This type of cloud entry and the electrostatic interactions between the wire antenna and its surroundings were simulated and are shown in Figure 13, Figure 14, Figure 15, Figure 16, Figure 17, Figure 18 and Figure 19 (antenna dimensions as presented in the beginning of Section 3; L = 140 m). The initial electric field strength of the antenna is 0, as it is assumed that the antenna lost its electric charge during the stay at higher altitudes with higher air conductivity.

The residual electric field strengths, remaining during the landing, differ between the cloud types and interaction models, but rarely exceed ±500 V/m. The five-times higher vertical velocity decreased the maximum electric field values reached in most cases in both models (while still the ‘dynamic’ model provides values one degree of magnitude higher than the ‘static’ approach)—this mechanism can be extrapolated for even higher, airplane-typical velocities, which would result in even lower differences between the total electric field strength and the external electric field strength—proving that the fast-moving aircraft is less prone to accumulating a large amount of electric charge in comparison with a slowly moving aerostat. For the Ns clouds, the cloud re-entry starts at the altitude, with higher conductivity than in the Sc cloud cases, thus preventing the electric field strength coming from the charging antenna to develop a significant difference between itself and the external electric field.

Table 1 presents the values of residual electric field strengths present at landing for different re-entry cases. For the dynamic cases, at the exits from the storm clouds, the lower air conductivity with different mechanisms of charge accumulation allows a significant residual electric charge to be present that shall discharge during the landing—a similar case to the LZ129 ‘Hindenburg’ disaster, which traveled through storm clouds prior to the landing attempt at Lakehurst [10].

The maximum values of the electric field strength reached on all the presented plots are significantly lower than the values of the corona breakdown electric field strengths, as mentioned in Section 2.1—this, however, does not necessarily mean that the corona is not likely to appear on the wire, as the presented electric fields have been calculated at a fixed distance r of 1 m—in the local zones closer to the surface of the wire, the electric field strength may reach the corona breakdown value. This is most likely in the approximate altitude ranges read from the plots (common for given model and flight stage):

- 100–800 m—Sc, ‘static’ model, ascend and re-entry phases,
- 900–1300 m—Sc, ‘dynamic’ model, ascend phase,
- >200 m—Sc, ‘dynamic’ model, re-entry phase,
- 4–7 km—Ns, ‘static’ model, ascend and re-entry phases,
- 8–10 km—Ns, ‘dynamic’ model, ascend phase,
- 0.1–2 km—Ns, ‘dynamic’ model, re-entry phase.

Both presented electrification models show increased charge gain on the antenna wire, despite the included functions of charge loss and, for the descent phases, increased velocity—this remains consistent with the influence of the electrification processes that drive the evolution and maturing of the clouds on other objects that appear in their proximities or insides [17]. The electric charge loss, depicted as faster at higher altitudes and slower at lower altitudes, due to changing conductivity of the air, can be related to the reported higher occurrence of lightning strikes appearing at lower altitudes on the aircraft [9]—as the aircraft is subjected to higher electric charges facilitating the formation of lightning.

The model that is considered most correctly describing the actual antenna wire electrification processes when passing through the clouds and their proximities is the ‘dynamic’ model, as it includes the expected (at given velocities of the balloon mission) influence of the antenna on the cloud’s electrical structure as a sort of an artificial disturbance. The ‘static’ model was built on simpler formulas which rely directly on the electrification processes in the clouds and the atmosphere, yet do not include the functioning of the cloud as a uniform, self-equalizing system—this appears as a crucial assumption for modeling electrostatic interactions which do resemble those appearing in reality in maturing charged clouds.

As both models did not possess an implemented breakdown/lightning-strike condition (this depends on the local structure of the cloud, which varies for every existing cloud), the model that showed the highest possibility of the occurrence of breakdowns is also the dynamic one, as in this model, only the electric field strength derivatives inside the clouds appear sufficiently large to sustain the rapid electrification after a possible discharge—a widely reported property of a highly electrically active cloud [17], imperceptible in the static model.

The dynamic model results may be compared to the experimental results, which concentrated on the monitoring of corona appearing on the balloon-borne wire antenna of the same dimensions as in the simulations, traveling directly through a warm storm front.

## 4. Experimental Flight through Storm

**Figure 20:**
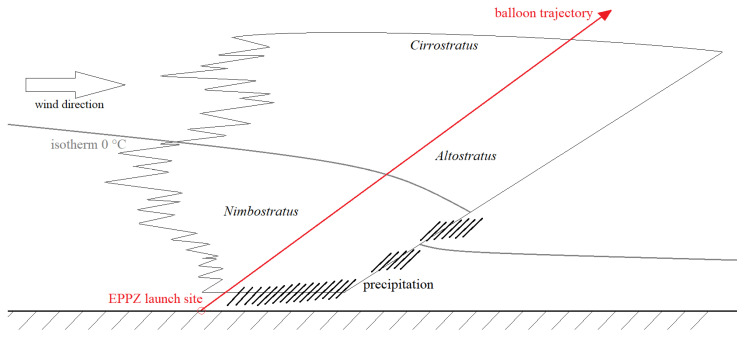
The schematic of the balloon mission’s trajectory through the storm front.

**Figure 21:**
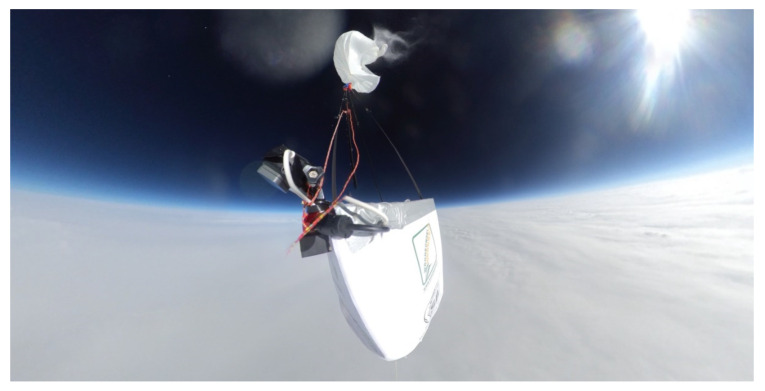
The view from the 360° camera affixed on the main gondola’s lid at the moment of the balloon burst at the maximum reached altitude. Below the gondola, the upper surface of the encountered storm front can be seen. The visibility inside the storm front dropped to single meters.

The research stratospheric balloon flight with one of the objectives to monitor the electrical interactions with the surrounding atmosphere took off from the Przasnysz Airfield (code: EPPZ) in Poland on 12 June 2021. A Styrofoam-made main gondola (cube with 0.3 m–long edge), suspended under a parachute and a latex helium-filled balloon, supported a 140 m–long tape antenna (dimensions and composition the same as described in the previous sections) with an additional polyethylene tether along it to reduce the mechanical strains; an extra radiosonde on a 2 m–long plastic tether was affixed below it, and a digital voltage meter was connected to the wire in the middle of its length (the meter failed to operate and delivered no data). The mission was launched shortly after a warm storm front passed above the airfield, allowing it to directly cross the Nimbostratus, Altostratus, and Cirrostratus cloud layers—as shown in Figure 20. Figure 21 presents the view from a 360° camera affixed on the lid of the main gondola at the maximum altitude reached (20,326 m). The hardware used to detect the corona consisted of a radio receiving circuit, equal to those used in mobile broadcasting receivers TENTO/KU-Zavod Neywa 402 [29], operating with a ferrite core antenna polarized vertically (covering the zone of the occurrence of possible corona, discharges, and streamers between the trailed wire and its surroundings). The operating frequency was set at 144 kHz, with ±4.5 kHz bandwidth of amplitude modulation detection; the frequency was chosen as a largely interference-free, positioned within the receiver’s range, below the AM broadcasting transmissions (with the first one, Antena Satelor from Romania, at 153 kHz) and above the numerous longwave teleswitches and time signals. The total payload of the balloon mission did not exceed 3.5 kg (categorized as a light atmosphere-sounding balloon).

The balloon mission landed safely on a local field, with the antenna fully stretched, proving that it traveled during the re-entry phase in the desired vertical position—the aluminum tape-like wire was, however, broken in multiple places. The avionics of the gondola remained intact, and the audio files from the receiver’s recorder were successfully retrieved for analysis.

## 5. Flight Results and Comparison with Theoretical Models

**Figure 22:**
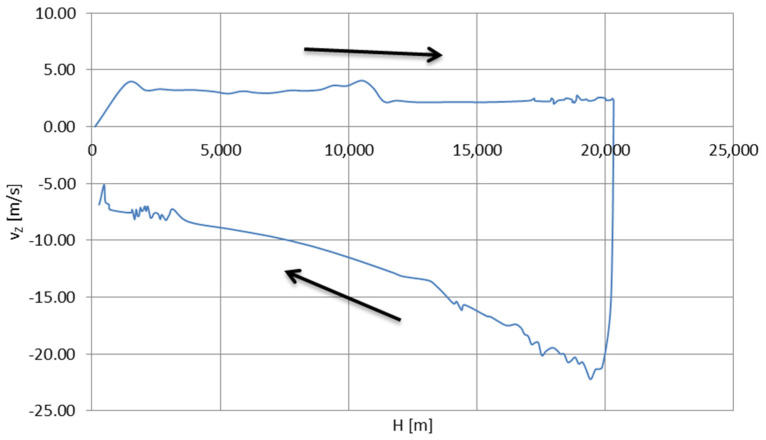
The vertical velocity of the balloon mission plotted against the altitude; the arrows indicate the direction of velocity change with the altitude values (the velocity dropped rapidly at the maximum altitude—the effect of balloon burst and the beginning of the re-entry phase).

Figure 22 presents the values of the ascend/re-entry velocities, *v_Z_* (m/s), obtained from the balloon system’s navigation system operating in the APRS network. The values of the *v_Z_* velocity of 3 m/s for the ascend cases and 15 m/s for the re-entry cases were taken directly from this data (with the latter velocity taken as an integer average over a larger altitude span).

**Figure 23:**
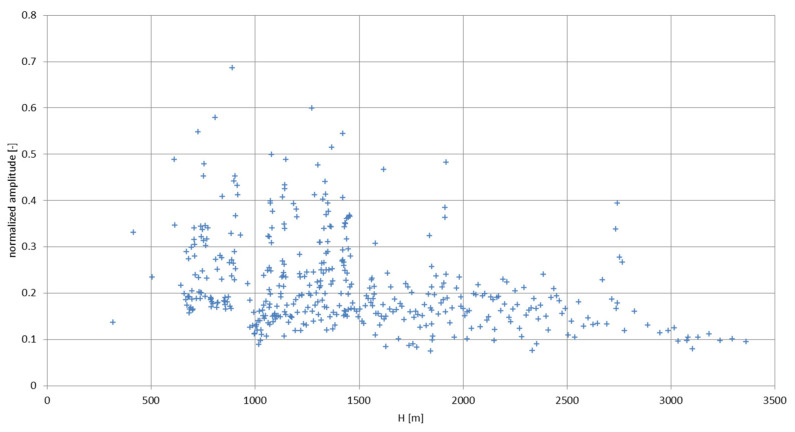
The recorded corona peaks extracted from the audio file.

Figure 23 shows the signal peaks extracted from the recorded longwave audio file. The phenomena appear between the altitude of 319 and 3363 m only; the receiving circuit has been operating for a short time before the launch, and the peaks did not appear in that period. It has been found that the peaks correspond to the corona electrical discharges [13,30], originating in the lower part of the storm front—the Ns cloud (as in Figure 20). Two distinct regions of peaks of different intensities can be noticed—the more intense lower region corresponds to the part of the cloud subjected to charge differentiation and electric field growth due to the rainfall [17]. Two larger peaks above 1600 m most likely correspond to the local irregularities in total electric field—local regions of higher concentration of electric charge.

**Figure 24:**
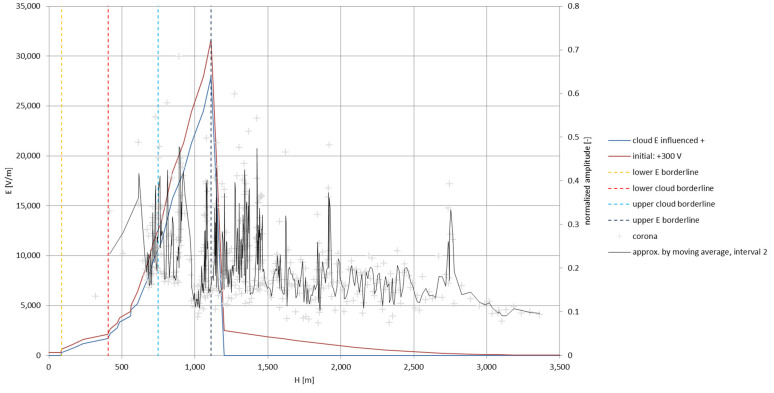
The comparison of the corona discharges plotted as a 2nd-order moving-average spline with the dynamic electrification of the airborne antenna moving through a positively polarized Sc cloud.

The higher concentration of corona discharges is directly linked to the increase of the electric field intensity around the balloon-borne antenna above a certain threshold value; the electric field strength, in order to excite the discharges, has to elevate itself rapidly in time—this property is manifested by the previously described dynamic model of electrification. The low altitude of the storm cloud during the experimental flight does not correspond to the lower cloud borderlines from the exemplary cases of the Ns clouds from Sankt Petersburg, Kyiv, and Tashkent due to local/climatic differences. As a best altitude match, Figure 24 presents the total electric field strength influenced by the antenna in the positively polarized Sc cloud (as in Figure 5b), in comparison with the corona discharges plotted in the form of a second-order moving-average function (splined values calculated as arithmetic average over two adjacent values). The most intense experimental corona region clearly corresponds to the zone where the highest calculated values of the total electric field strength inside the cloud are expected to appear, according to the calculations from the ‘dynamic’ model; the existence of the latter corona peaks can be attributed to, as previously mentioned, encountered cloud/charge irregularities at higher altitudes.

The experimental corona discharge data did not deliver an explicit information on the values of the electric current that flew during the discharges. For a qualitative insight, from ground measurements on high metallic or lightning-rod-equipped objects [14], an average order of magnitude of such currents can be attributed by multiplying the normalized amplitude values from Figure 23 with 10^3^, giving the approximate discharge currents in amperes. With the average discharge time of 1 millisecond, the average electric charge transfer in coulombs can be defined by integrating in time domain the current impulses [14]. Table 2 presents the calculated values for three altitude ranges; the informative orders of magnitude of the charge transfers (C) and discharge currents (A) are calculated as log_10_x, with x being the aforementioned values. Based on the previous assumption for currents’ definition (based on ground data from Reference [14]), it can be seen that the order of magnitude of the total charge transfer via corona discharges for the experimental balloon-borne antenna of 140 m in length reaches the values close to the total charge values for a single storm cloud [17]—an effect of artificially induced intense electrification, which can be explained by the conductivity-based interaction of the slowly moving antenna with the cloud—the dynamic model of electrification.

## 6. Discussion

The dynamic model of balloon-borne antenna electrification in every considered cloud case has produced a single maximum peak of electric field strength (global maximum). The experimental corona discharge plot shows multiple peaks instead of one, considering that the discharges occur at the maximum electric field strength—this difference is the effect of the exemplary value of *r* = 1 m at which the calculations were performed (as there is no solid second electrode in real conditions). Multiple discharge peaks show that the corona threshold value for the electric field strength is reached more rapidly and more often—an effect of the intense electrifying processes and, as the antenna is fully immersed in the charging medium with no separation/insulation, physically lower values of the maximum distance, *r*, between the antenna wire and surrounding charged humid air concentrations.

The plotted electric field strengths for both models and all cloud cases depict the electric field strength reached around the antenna wire in the moment the wire passes through the cloud/space of certain properties; after the antenna moves up and forward, the electric field strength either drops down in time to its previous value or evolves with the conventional evolution processes with the cloud. It should be, therefore, theoretically possible to influence the formation and evolution of clouds (by affecting their electric fields) by inserting into them a slow-moving, highly conductive disturbance in the form of a long antenna wire. Based on the qualitative analysis of the corona discharge currents and total charge flows, it can be expected that, from the perspective of the cloud, the electrifying effects of the antenna wire on the cloud structure cannot be treated as negligible; in an extreme case, the cloud may try to maintain its equilibrium by ejecting a certain electric charge upward—an ephemeral effect often known as the TLE (Transient Luminous Event), which is observed naturally only above highly electrified clouds.

The calculation of the discharge currents from the recorded corona peaks can be performed accurately if a Pearson coil is employed as a receiving end of the recording system. This type of coil is, however, unadapted for light stratospheric balloon missions because of its small dimensions and very high density—this, from the point of flight safety, automatically elevates the balloon mission class to heavy, which demands more elaborated navigation and in-flight control; moreover, the majority of the payload available is dedicated to the coil only.

It has been reported that the corona discharges are excited on the entire length of the antenna wire [21]. Each discharge is a high-temperature phenomenon which does not leave the substrate material undamaged—microcraters from the discharge entry/exit points are able to mechanically weaken the material, facilitating the propagation of cracks during the mechanical strains in the re-entry phase—this is most likely one of the contributing factors to the fact that the experimental antenna wire, subjected to intense corona in the storm cloud, landed with its aluminum part separated into pieces. As the entire wire is subjected to corona, the electric charge lost in each corona peak comes from the entire conductive part of the wire—these current peaks pass also through the antenna transformer, creating high-voltage peaks endangering the transmitter—especially if it is based on high-voltage-sensitive MOSFET transistors. Therefore, a set of protective measures must be undertaken to ensure the safe operation of the transmitter. The most intuitive way is to disconnect the transmitter from the antenna and operate it above the clouds only, and this, however, limits the research capabilities of such transmitting systems. Any ferrite snubbers provide sufficient protection only if employed as a part of a larger protective system, relying mainly on automatic overload detection [31]—provided the total system is not too heavy and dense for a light-class balloon mission and not too payload-consuming for a heavier mission. A passive and effective system protecting the transmitter is the low efficiency of the antenna transformer, which can assure acceptable high-voltage risk levels for the semiconductor-based transmitter, but with lower transmitting efficiency—to be overcome by either increased transmitter power or increased sensitivity of the receivers.

The corona discharges, however, can be concentrated around mechanical structures which provide high electric charge density—spikes, single wires, or wire bundles (similar to those shown in Figure 1), constituting an electrostatic discharger—a safe location for rapid charge loss without the risk of mechanical damage to the antenna wire and, if the charge loss is continuous and rapid enough, low corona current peaks not endangering the transmitting circuit. An active solution for the reduction of the electric charge—as in active inductive dischargers used in industrial applications [24]—is unfeasible, as the system is a floating-earth conductor, with no permanent grounding.

## 7. Conclusions

This paper presented two theoretical approaches to the description of the electrification of a long balloon-borne wire antenna traveling through different types of clouds. Based on the experimental flight through a storm cloud with intense corona discharges, the more accurate electrification model was chosen (the ‘dynamic’ one), with the differences between the model and the experiment indicated. The electrification models can be refined with more detailed formulas describing the electrification processes in various atmosphere layers for different antenna geometries. A set of basic requirements for the antenna system was defined in order to reduce the risk of transmitter overload and antenna damage due to ongoing corona. An open problem is the accurate theoretical modeling of the ‘2nd electrode’ for atmospheric electrification processes and discharges; an engineering task would be to create a sufficiently lightweight and safe method for the discharge current measurements onboard a stratospheric balloon mission that is not based on a too-dense and too-heavy Pearson coil.

## Figures and Tables

**Table 1:** The residual electric field strengths calculated for the moment of landing for different charge accumulation cases at the re-entry velocity of 15 m/s.

‘STATIC’ CASE		‘DYNAMIC’ CASE		Difference
Cloud Type	E (V/m)	Cloud Type	E (V/m)	(V/m)
Sc polarization positive	28.46	Sc polarization positive	580.56	552.10
Sc polarization negative	−12.72	Sc polarization negative	−229.61	−216.89
Sc charge positive	10.37	Sc charge positive	333.60	323.23
S*c* charge negative	68.26	Sc charge negative	602.99	534.73
Ns Sankt Petersburg	4.53	Ns Sankt Petersburg	72.97	68.44
Ns Kyiv	−0.48	Ns Kyiv	243.33	243.81
Ns Tashkent	−20.40	Ns Tashkent	2394.72	2415.12

**Table 2:** The calculated qualitative values of the charge transfer and discharge current.

Entire Region (319–3363 m)	
charge transfer (C)	order of magnitude (log_10_x)
46.592	1.668
average current (A)	order of magnitude (log_10_x)
107.852	2.033
**Large Charge Region (319–1500 m)**	
charge transfer (C)	order of magnitude (log_10_x)
32.469	1.511
average current (A)	order of magnitude (log_10_x)
122.065	2.087
**Small Charge Region (1500–3363 m)**	
charge transfer (C)	order of magnitude (log_10_x)
14.123	1.150
average current (A)	order of magnitude (log_10_x)
85.078	1.930

## Data Availability

The new data presented in this study are available on request from the corresponding author.
